# Interaction of local solidification and remelting during dendrite coarsening - modeling and comparison with experiments

**DOI:** 10.1038/s41598-017-17857-2

**Published:** 2017-12-19

**Authors:** Qingyu Zhang, Hui Fang, Hua Xue, Shiyan Pan, Markus Rettenmayr, Mingfang Zhu

**Affiliations:** 1 Jiangsu Key Laboratory for Advanced Metallic Materials, School of Materials Science and Engineering, Southeast University, Nanjing, 211189 China; 2 School of Materials Science and Engineering, Nanjing University of Science and Technology, Nanjing, 210094 China; 3 Otto Schott Institute of Materials Research, Friedrich Schiller University, Löbdergraben 32, Jena, 07743 Germany

## Abstract

The microstructural evolution of dendrite coarsening during isothermal holding is simulated using a quantitative cellular automaton (CA) model involving the mechanisms of both solidification and melting. The present model encompasses the essential aspects of thermodynamics and kinetics, particularly the evolution/influence of composition, temperature, and curvature, leading to valid simulations of simultaneous solidification and melting. Model validation is performed through a comparison of the CA simulations with analytical predictions for a liquid pool migrating in the mushy zone of a SCN–0.3 wt.% ACE alloy due to temperature gradient zone melting. The model is applied to simulate the microstructural evolution of columnar dendrites of a SCN–2.0 wt.% ACE alloy during isothermal holding in a mushy zone. The simulation results are compared with those of a previous CA model that does not include the melting mechanism under otherwise identical conditions. The role of melting for dendrite coarsening is quantified, showing how the melting influences the coarsening process. The present model effectively reproduces the typical dendrite coarsening features as observed in experiments reported in the literature. The simulations reveal how local solidification and melting stimulate each other through the complicated interactions between phase transformation, interface shape variation, and solute diffusion.

## Introduction

Understanding microstructural evolution during solidification is the prerequisite for controlling microstructures and thus vital for achieving desired properties of castings. Dendrites are certainly the most frequently observed solidification microstructure of metallic alloys. In most solidification processes at low and moderate cooling rates, dendrite coarsening in mushy zones has been recognized as an unavoidable phenomenon that influences microstructures and thereby the properties of the fully solidified materials significantly^1,2^. Owing to the importance of dendrite coarsening for both academic value and practical application, the associated studies have received persistent scientific interest for fundamentally understanding the behavior of dendrite coarsening during solidification or under isothermal conditions.

Lifshitz and Slyozov^3^ and Wagner^4^ proposed that for the self-similar coarsening of spherical particles, the average particle size evolves according to the power-law relation

1 $${\bar{R}}^{3}(t)-{\bar{R}}^{3}(0)={K}_{LSW}t$$

where $\bar{R}(t)$ is the average particle radius at time *t*, $\bar{R}(0)$ is the average particle radius at the start of self-similar coarsening, and *K*_*LSW*_ is the coarsening constant. For dendrite coarsening, the specific surface area, defined as the total solid/liquid (S/L) interfacial area per unit volume of a solid-liquid mixture, *S*_*V*_, is commonly used as the characteristic length. Marsh and Glicksman^1^ suggested that for diffusion-limited coarsening in conserved volume fraction systems, the decay of *S*_*V*_ has the form that is analogous to Eq. (1)

2 $${S}_{V}^{\,-3}(t)-{S}_{V}^{\,-3}(0)={K}_{C}t$$

The main mechanisms of dendrite coarsening have been described to be the dissolution of small arms to the benefit of adjacent larger arms, gradual movement of the interdendritic groove bases towards the arm tips, coalescence of arms near the tips, leading to the entrapment of liquid in the solid, and dendrite arm fragmentation^5–10^. Based on the Gibbs-Thomson equation at the S/L interface and solute conservation, analytical models were developed to predict the time-dependent positions of the melting arm tip and the advancing interdendritic groove base^5–7^. Those analytical models assumed that the dendrite arms retain the simplified geometries of cylinders with hemispherical tips during coarsening; the regions of local remelting and solidification were predefined in a simplified environment. In addition, the solute was assumed to diffuse between the shrinking arm tip and the growing interfaces by solving a one-dimensional solute flux equation with an approximate diffusion length. Accordingly, the analytical models yield rather semi-quantitative estimates for the kinetics of dendrite coarsening, but do not deal explicitly with the different mechanisms that operate concurrently.

Extensive experimental studies have been carried out to investigate the processes of dendrite coarsening by *post-mortem* analyses of samples quenched from mushy zones^11–14^ or by *in situ* observations using transparent alloys^15^ and synchrotron-based X-ray tomography^9,16–20^. The coarsening mechanisms as stated above were clearly identified from the *in situ* observations, and those mechanisms were commonly found to occur simultaneously^9^. In order to investigate the kinetics of dendrite coarsening, interface shape and specific surface area, *S*_*V*_, are quantified as a function of time^9,11–13,16,17^. The majority of those experimental studies revealed that *S*_*V*_ follows a *t* ^−1/3^ power law during isothermal holding^11–13,16,17^, even though the solid-liquid mixtures were not evolving self-similarly^11,12^. Fife *et al*.^16^ examined the global dynamics of isothermal coarsening of Al–Cu dendritic microstructures and found that the interface velocities depend on mean curvatures, but exhibit large dispersions for a single value of mean curvature, indicating that there is a strong effect of the local environment on the evolution of the S/L interface. The phenomenon of dendrite arm fragmentation has also been observed in continuous solidification^21^ and isothermal holding^14,15^ in mushy zones. Cool and Voorhees^14^ performed isothermal coarsening experiments in mushy zones using Pb-Sn alloys aboard the International Space Station (ISS). Microstructural evolution and dendrite fragmentation were determined using three-dimensional reconstructions. The number of fragments scaled by ${S}_{V}^{\,-3}$ was found to be independent of time. All of these experimental studies have provided a lot of meaningful information about the mechanisms that occurred during the processes of dendrite coarsening. Note that however, for the quantitative analysis of microstructural evolution in mushy zones, it is critical to accurately measure the variations of local temperature, composition, and interfacial curvature with time. Yet, at present it is still challenging to conduct real-time accurate measurements of those quantities even using *in situ* high speed synchrotron tomography^16–22^. During those experiments, direct measurement of the sample temperature is not possible because of encapsulation^17^; the measurement of composition fields in mushy zones is still on a semi-quantitative or qualitative level^21,22^. Moreover, after acquiring X-ray projections of the samples, complex procedures of three-dimensional (3-D) reconstruction and image processing are required for quantifying the volume, surface area and surface curvatures^16–20^.

Over the recent decades, computational modeling has developed rapidly and presents considerable potential for providing a complete time-dependent description of evolving arbitrarily complex interfacial morphologies. It has thus become a powerful and indispensable tool in studying microstructural evolution during various phase transformations.

Neumann-Heyme *et al*.^23^ proposed a numerical model of a single secondary arm to simulate the dynamics of dendrite sidebranch detachment from the main stem. The results showed that pinch-off only occurs in limited ranges of geometrical parameters and cooling rates and is generally bounded by sidearm retraction and coalescence regimes.

Phase-field (PF) simulations have been performed to investigate dendrite coarsening^24–28^ and dendrite fragmentation^29^. In those simulations, the evolution of dendrite morphology during holding in mushy zones was appropriately reproduced. Nevertheless, comprehensive studies of the complicated interactions among the kinetics of local solidification/melting, interfacial curvature, and solute diffusion have so far remained scarce. In addition, PF models inherently involve both solidification and melting mechanisms, and the role of melting cannot be separated and quantified.

The cellular automaton (CA) method can handle complex topology changes and has some attractive advantages such as the simplicity of formulation and computational convenience when implemented to solve phase transition problems with an acceptable computational efficiency. Extensive efforts have been dedicated to the development of various models based on the CA method to simulate a wide variety of solidification microstructures^30–39^. Recently, Chen *et al*.^30^ and Zinoviev *et al*.^31^ proposed mesoscale CA models that involve melting and growth of grains in mushy zones for the simulation of grain structure formation in the arc-welding and laser additive manufacturing processes. To the best of our knowledge, however, all existing microscale CA models only consider solidification, but neglect melting^32–39^, and thus are unable to appropriately describe dendrite coarsening, for which melting is by no means negligible.

Thus, an advanced knowledge base has been put up regarding dendrite coarsening by theoretical analysis, experimental studies, and simulations. It is well accepted that a remelting/resolidification mechanism is responsible for dendrite arm coarsening^1,40^, and that solidification and melting stimulate each other^1^; the kinetics of local melting/solidification is not only influenced by the local interfacial curvature, but also by the neighboring morphologies^16^. Nevertheless, there is still a lack of detailed data to clearly visualize how solidification and melting stimulate each other and how the local environment influences the kinetics of local solidification/ melting. In addition, as stated above, so far the contribution of melting on dendrite coarsening has in its complexity not been quantified.

In the present study, a two-dimensional (2-D) quantitative CA model is proposed for the simulation of microstructural evolution involving both solidification and melting. The model allows visualizing the ongoing evolution of local equilibrium composition and actual composition. The proposed model is adopted to simulate dendrite coarsening in an isothermal mushy zone of SCN–ACE alloys. The interactions between local solidification/melting, interfacial shape, and solute diffusion are analyzed in detail by means of a direct comparison of the local equilibrium liquid composition with the actual liquid composition. The role of remelting on dendrite coarsening is also discussed by comparing the results obtained by the present model and by a previous CA model that does not include melting.

### Model description, governing equations and numerical algorithm

Aim of the present study is to simulate microstructural evolution of dendrite coarsening in a mushy zone. The solute transport is considered to be controlled solely by diffusion. Under the assumption of local equilibrium at the interface, the phase transformation is simulated symmetrically for solidification and melting at low migration velocities of the S/L interface, i.e. the migration of the S/L interface is governed by solute diffusion and interface curvature, while the effect of interface kinetics leading e.g. to solute trapping is neglected^41^. The computation domain is divided into uniform square cells (mesh units). Each cell is characterized by several variables, including composition (*C*), solid fraction (*f*_s_), temperature (*T*), crystallographic orientation (*θ*). The state of a cell can be liquid (*f*_s_ = 0), solid (*f*_s_ = 1), or S/L interface (0 < *f*_s_ < 1). The temperature field in the domain is either an imposed time-invariant temperature gradient, or set to be uniform at a given temperature between the solidus and liquidus temperatures. The driving force for the migration of the S/L interface is determined using a local composition equilibrium approach^34^ that calculates the kinetics of solidification/melting according to the difference between the local equilibrium composition and local actual composition. The governing equations and numerical algorithms for calculating the kinetics of the S/L interface migration, the interface weighted curvature, the crystallographic anisotropy, and the composition field are described in detail below.

According to the local thermodynamic equilibrium at the S/L interface, the local equilibrium liquid composition,$\,{C}_{l}^{eq}$, is calculated by

3 $${{C}}_{{l}}^{{e}{q}}{=}{(}{{T}}^{\ast }{-}{{T}}_{{m}}{)}{/}{{m}}_{{l}}{+}{\Gamma }{{K}}_{{w}}{/}{{m}}_{{l}}$$

where *T* ^*^ is the local interface temperature, *T*_m_ is the melting point of the pure solvent, *m*_*l*_ is the liquidus slope, *Γ* is the Gibbs-Thomson coefficient, *K*_*w*_ is the weighted curvature that is calculated from the gradient of the solid fraction at the S/L interface and connected with the anisotropy of surface energy by

4 $${K}_{w}={({(\frac{{\rm{\partial }}{f}_{s}}{{\rm{\partial }}x})}^{2}+{(\frac{{\rm{\partial }}{f}_{s}}{{\rm{\partial }}y})}^{2})}^{-\frac{3}{2}}\times (2\frac{{\rm{\partial }}{f}_{s}}{{\rm{\partial }}x}\frac{{\rm{\partial }}{f}_{s}}{{\rm{\partial }}y}\frac{{{\rm{\partial }}}^{2}{f}_{s}}{{\rm{\partial }}x{\rm{\partial }}y}-{(\frac{{\rm{\partial }}{f}_{s}}{{\rm{\partial }}x})}^{2}\frac{{{\rm{\partial }}}^{2}{f}_{s}}{{\rm{\partial }}{y}^{2}}-{(\frac{{\rm{\partial }}{f}_{s}}{{\rm{\partial }}y})}^{2}\frac{{{\rm{\partial }}}^{2}{f}_{s}}{{\rm{\partial }}{x}^{2}})(1-15e\,\cos (4(f-{\partial }_{0})))$$

where *f*_*s*_ is the solid fraction of an interface cell, *ε* is the degree of anisotropy of the surface energy, *θ*_0_ is the angle of the preferential growth direction with respect to the *x*-axis, *φ* is the growth angle between the normal to the interface and the *x*-axis, and it is calculated by

5 $$\phi =\arccos (\frac{\partial {f}_{s}/\partial x}{{({(\partial {f}_{s}/\partial x)}^{2}+{(\partial {f}_{s}/\partial y)}^{2})}^{1/2}})\,$$

According to the solute equilibrium condition at the interface, during a given time step interval, Δ*t*, the increment in the solid fraction in an interface cell, Δ*f*_*s*_, is calculated by

6 $${\rm{\Delta }}{f}_{s}=g({C}_{l}^{eq}-{C}_{l}^{\ast })/({C}_{l}^{eq}(1-k))$$

where ${C}_{l}^{eq}$ is defined in Eq. (3), and ${C}_{l}^{\ast }$ is the local actual liquid composition at the S/L interface obtained from the mass transport calculation, *k* is the partition coefficient. Eq. (6) was originally proposed for simulating dendrite solidification^34^ based on the classical model of solute conservation at the interface^32^. It has been validated that after the dendrite reaches the steady-state growth, the solute conservation at the moving S/L interface is preserved, indicating that the model given by Eq. (6) recovers the Stefan condition during the steady-state stage of phase transformation^34^.

In Eq. (6), *g* is a geometrical factor that is introduced to reduce the artificial anisotropy caused by the CA square cell, defined by

7a $$g=\,\min ((\sum _{m=1}^{4}{S}_{m}^{I}+\frac{1}{\sqrt{2}}\sum _{m=1}^{4}{S}_{m}^{{\rm{II}}})/2,1)$$

7b $$\mathrm{solidification}:{S}^{I},{S}^{{\rm{II}}}=\{\begin{array}{c}\,0({f}_{s} < 1)\\ \,1({f}_{s}=1)\end{array}$$

7c $$\mathrm{melting}:{S}^{I},{S}^{{\rm{II}}}=\{\begin{array}{c}\,0({f}_{s} > 0)\\ \,1({f}_{s}=0)\end{array}$$

where *S* ^*I*^ and *S* ^*II*^ are the states of the nearest and second-nearest neighboring cells. The value of Δ*f*_*s*_ obtained from Eq. (6) determines the migration direction of the S/L interface, particularly toward the liquid phase (solidification) if Δ*f*_*s*_ > 0, or toward the solid phase (melting) if Δ*f*_*s*_ < 0. Apparently, the kinetics of solidification and melting calculated by Eq. (6) incorporates the effects of interface temperature, curvature, and solute diffusion through ${C}_{l}^{eq}$ and ${C}_{l}^{\ast }$. During solidification or melting, solute partitioning at the S/L interface is considered according to ${C}_{s}^{\ast }\,=\,k{C}_{l}^{\ast }$, where ${C}_{s}^{\ast }$ and ${C}_{l}^{\ast }$ are the local interface compositions in the solid and liquid phases, respectively. The mean concentration of each cell is calculated using the lever rule *C* = *C*_*s*_ *f*_*s*_ + *C*_*l*_(1 − *f*_*s*_). The governing equation for calculating solute partitioning and diffusion within the entire domain can be written as

8 $$\frac{\partial {C}}{\partial {t}}=\nabla \cdot (D({f}_{s})\nabla (C/p({f}_{s})))$$

where *p*(*f*_*s*_) is associated with the solid fraction, calculated by *p*(*f*_*s*_) = *kf*_*s*_ + (1 − *f*_*s*_), *D*(*f*_*s*_) is the diffusion coefficient depending on the solid fraction, calculated through *D*(*f*_*s*_) = *kD*_*s*_ *f*_*s*_ + *D*_*l*_(1 − *f*_*s*_), where *D*_*s*_ and *D*_*l*_ are the solute diffusivities in the solid and liquid phases, respectively.

Equation (8) is derived based on the mass conservation and solute equilibrium at the S/L interface. The term, ${\rm{\partial }}{C}/{\rm{\partial }}{t}$, on the left-hand side of Eq. (8) includes the effect of solute partitioning during the liquid-solid phase transformation. Consequently, Equation (8) solves the problems of discontinuous solute concentration and solute partitioning at the S/L interface in a straightforward manner, and the entire computation domain can be treated as a single phase for the calculation of solute transport^42^. We have tested that during simulations the average composition in the domain is constant, indicating that the solute conservation at the S/L interface is maintained. Equation (8) is solved using the explicit finite difference method. The time step is determined by Δ*t* = Δ*x* ^2^/8*D*_*l*_, where Δ*x* is the cell size. Zero-flux boundary conditions are adopted on all walls of the computation domain.

The solution sequence of the equations and algorithms described above proceeds iteratively as follows:

(1) Initialize the simulation system with domain length, cell size, composition, and temperature fields.

(2) Calculate the equilibrium composition using Eq. (3) and curvature using Eqs (4) and (5).

(3) Calculate the increment in solid fraction using Eqs (6) and (7).

(4) Calculate the solute field by solving Eq. (8).

(5) Update the solid fraction field.

(6) Proceed to the next time step from step (2) until the simulation ends.

The physical properties of the SCN–ACE alloys studied in the present study are listed in Table 1 ^43^.

**Table 1:** Physical parameters of SCN-ACE alloys as used in the present work^43^.

Parameter	value
Gibbs-Thomson coefficient, *Γ*	6.48 × 10^−8^ °C·m
Diffusion coefficient in liquid, *D*_*l*_	1 × 10^−9^ m^2^/s
Diffusion coefficient in solid, *D*_*s*_	1 × 10^−12^ m^2^/s
Partition coefficient, *k*	0.1
Liquidus slope, *m*	−2.8 °C/wt.%
Melting point of the pure solvent, *T*_*m*_	58.081 °C

## Results and Discussion

### Model validation

**Figure 1:**
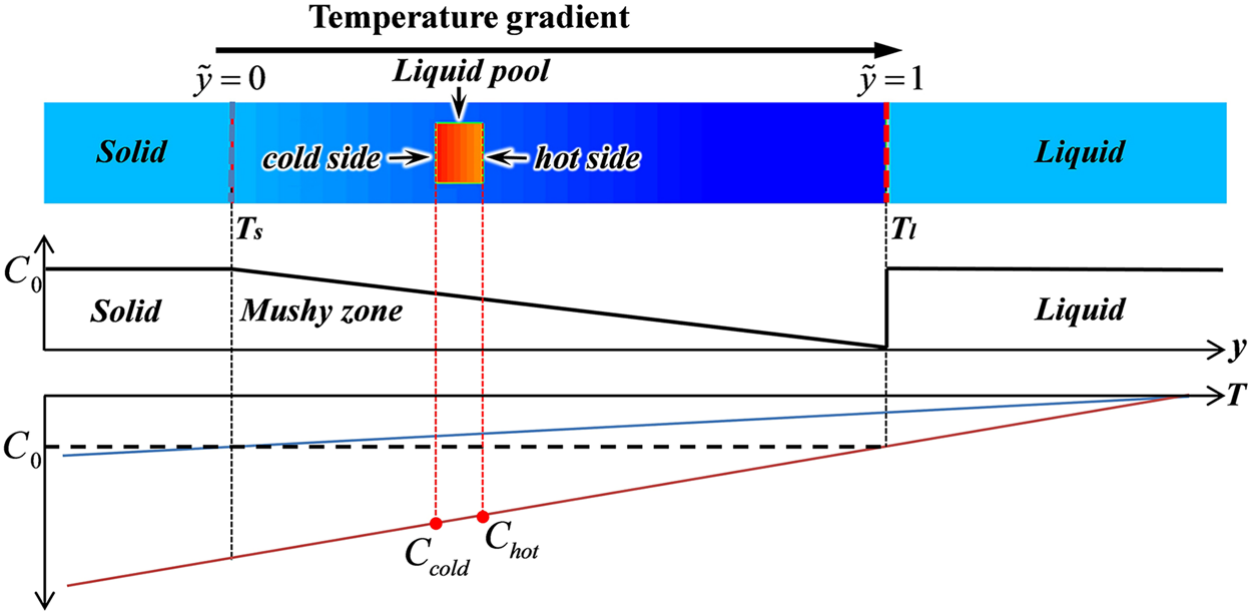
Schematic illustration of liquid pool migration in the solid matrix due to temperature gradient zone melting.

To validate the proposed CA model for the quantitative simulation of microstructural evolution during melting/solidification, the migration of a liquid pool in a temperature gradient is simulated and compared with the prediction of the analytical model. As illustrated in Fig. 1, in the presence of a temperature gradient, the adjustment of the temperature-dependent local equilibrium compositions at neighboring S/L interfaces establishes a composition gradient across the liquid pool. The composition gradient drives solute atoms to diffuse from the colder side with higher composition to the hotter side with lower composition. Solute enrichment at the hotter S/L interface leads to its melting, while solute depletion at the colder S/L interface causes resolidification. This melting/resolidification process results in liquid pool migration towards the high temperature direction, which is termed temperature gradient zone melting (TGZM)^40^.

Pan *et al*.^44^ proposed an analytical model for the prediction of a liquid droplet/pool migrating in a mushy zone due to TGZM for both static and dynamic conditions. In the static case without pulling, the dimensionless migration velocity of a liquid pool can be expressed as

9 $$\mathop{V}\limits^{ \sim }=\frac{d\mathop{y}\limits^{ \sim }}{d\mathop{t}\limits^{ \sim }}=\frac{1}{1-(1\,-\,{k})\cdot \mathop{y}\limits^{ \sim }}$$

where $\mathop{y}\limits^{ \sim }$ and $\mathop{V}\limits^{ \sim }$ are the dimensionless time-dependent position and migration velocity of the liquid pool, respectively. By solving Eq. (9), the dimensionless pool position is obtained as

10 $$\mathop{y}\limits^{ \sim }(\mathop{t}\limits^{ \sim })=(1-\sqrt{{(1-(1-k){\mathop{y}\limits^{ \sim }}_{0})}^{2}-2(1-k)\mathop{t}\limits^{ \sim }})/(1-k)$$

where $\mathop{t}\limits^{ \sim }$ and ${\tilde{y}}_{0}$ are the dimensionless time and initial pool position, respectively. Substituting $\mathop{y}\limits^{ \sim }(\mathop{t}\limits^{ \sim })$ calculated from Eq. (10) into Eq. (9), the dimensionless pool migration velocity, $\tilde{V}$, is obtained. Then, transforming $\tilde{t}$, $\tilde{y}(\tilde{t})$, and $\tilde{V}$ to dimensional variables through *t* = $\tilde{t}\cdot {l}_{T}^{2}/{D}_{l}$, *y* = $\tilde{y}\cdot$ *l*_*T*_, and *V* = $\tilde{V}\cdot {D}_{l}/{l}_{T}$, where *l*_*T*_ is the mushy zone length, the pool position, *y*, and migration velocity, *V*, as functions of time can be explicitly solved.

The CA simulation is performed for liquid pool migration of a SCN–0.3 wt.% ACE alloy in a static temperature gradient of *G*_*T*_ = 10 °C/mm. The domain consists of a 10 × 900 mesh with Δ*x* = 1.2 μm. The stationary solidus and liquidus are located at *y* = 60 μm ($\tilde{y}$ = 0) and *y* = 816 μm ($\tilde{y}$=1), respectively. As shown in Fig. 1, the compositions of the bulk solid phase at the left of the solidus, and the bulk liquid phase at the right of the liquidus, are uniform and equal to *C*_0_ = 0.3 wt.% ACE. The region between the solidus and liquidus is initialized as a solid matrix with a solid composition gradient following the solidus line. A rectangular liquid pool with a thickness of 12 μm and the composition, determined from the phase diagram according to the local temperature, is initially placed close to the solidus, *y* = 135.6 μm (${\tilde{y}}_{0}$ = 0.1). The position, *y*, and migration velocity, *V*, are calculated for the pool center.

**Figure 2:**
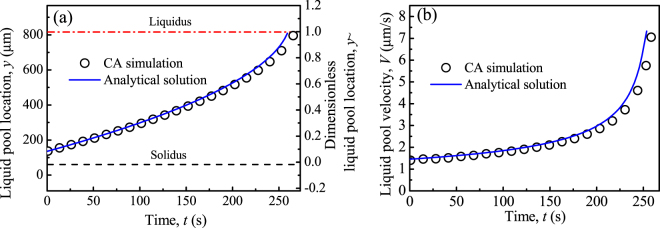
Comparison of CA simulation and analytical prediction regarding the time evolution of (**a**) location and (**b**) migration velocity of a liquid pool (${\tilde{y}}_{0}$ = 0.1) for a SCN–0.3 wt.% ACE alloy at *G*_*T*_ = 10 °C/mm.

Figure 2 presents the time evolution of pool location and migration velocity obtained by the CA and analytical models. It is found in Fig. 2a that the pool migrates towards the high temperature direction from the initial position to the liquidus position. It eventually reaches the (stationary) liquidus. As indicated in Eq. (9), the migration velocity increases with the dimensionless position, $\tilde{y}$. Thus, as the pool is approaching the liquidus, its migration velocity gradually increases with time as shown in Fig. 2b. The results presented in Fig. 2 show that the profiles obtained from the CA simulations agree well with the analytical solutions, demonstrating the validity of the proposed CA model for the quantitative simulation of S/L interface migration due to simultaneous melting/resolidification.

Recently, we also applied the proposed CA model to simulate liquid pool migration in the mushy zone with different pulling velocities and dendrite arm migration in a temperature gradient. The CA simulations agree well with the relevant analytical predictions and experimental measurements. Those validation results will be reported in an upcoming paper.

### Dendrite arm coarsening

**Figure 3:**
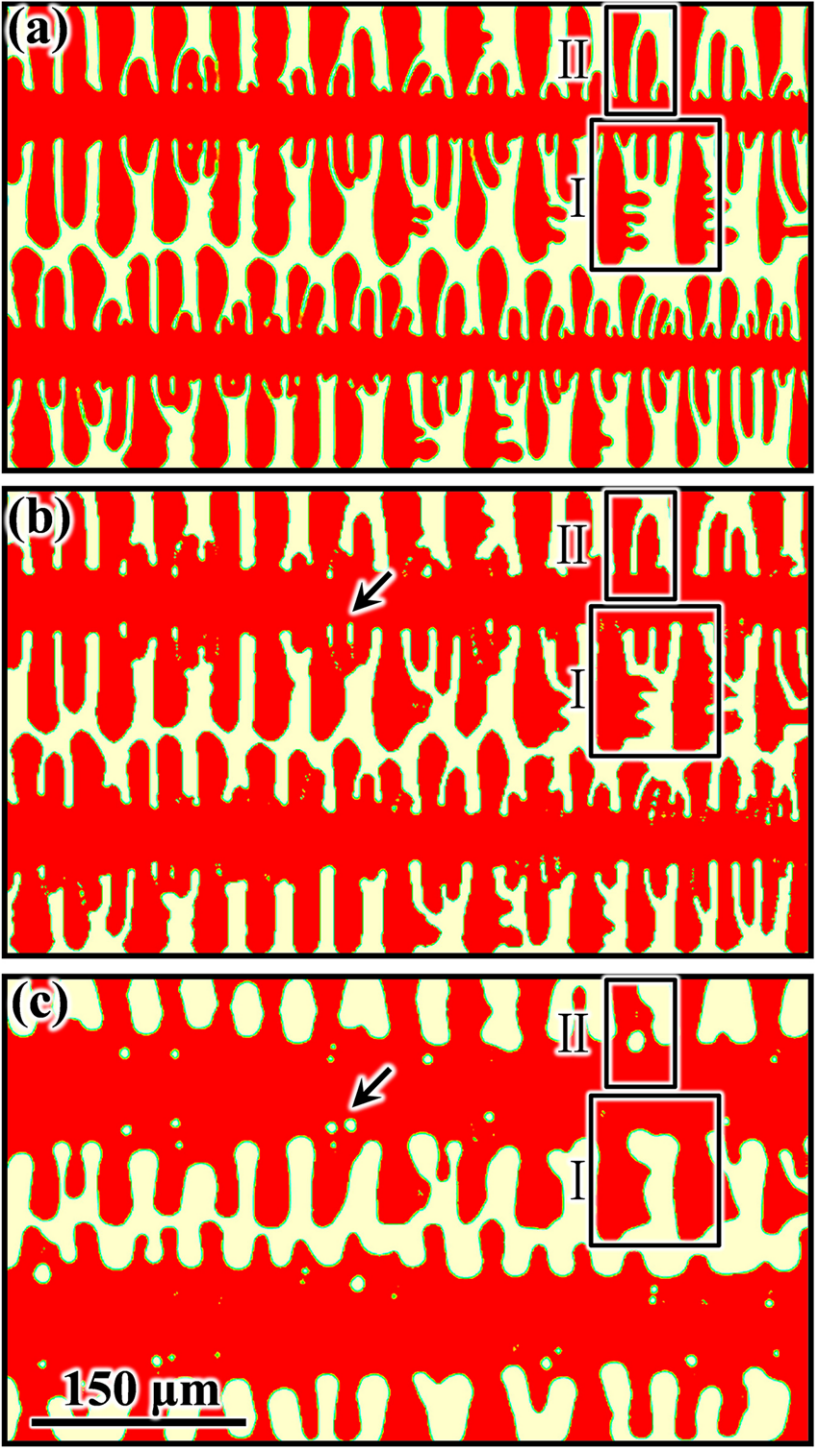
Simulated dendrite arm coarsening for a SCN–2.0 wt.% ACE alloy displayed in solid fraction: (**a**) starting microstructure; (**b**) and (**c**) microstructures simulated by the previous and present CA models, respectively, after isothermal holding in a mushy zone of 42.8 °C for 600 s. Detailed microstructural evolution of the columnar dendrites in (**c**) can be seen in Supplementary video 1.

The proposed model is applied to simulate the microstructural evolution of a SCN–2.0 wt.% ACE alloy during isothermal holding in a mushy zone with a uniform temperature. The computation domain consists of a 280 × 470 mesh with Δ*x* = 1.2 μm. The simulations are carried out in two stages. First, directional solidification is simulated using the previous CA model with *G*_*T*_ *= *7 °C/mm. At a solid fraction of 70.2%, the temperature in the domain was set to be homogeneously 42.8 °C throughout the calculation domain. After holding for a short time of ~0.2 s for stabilization, the starting microstructure at time *t*_*start*_ is obtained. Second, microstructural evolution during isothermal holding at 42.8 °C is simulated by both the previous and the present CA models. Figure 3 presents the simulated dendritic microstructures at time *t*_*start*_, and after holding at 42.8 °C for 600 s, as obtained from the two CA models. During isothermal holding, the solid fractions remain in the range of 69.8% ∼ 70.4%, i.e. in the range of 70.2% as calculated using the lever rule based on the equilibrium phase diagram of SCN-ACE alloys. As shown in Fig. 3, while the dendritic microstructures in both Fig. 3b and c are found to be coarser than the starting microstructure in Fig. 3a, the dendrite morphologies in Fig. 3b and c are remarkably different. For example, the primary dendrite stem in Fig. 3c is obviously thicker than that in Fig. 3b. Some small secondary and tertiary arms presented in Fig. 3a are still visible in Fig. 3b, but have completely disappeared in Fig. 3c (e.g. in the region indicated by Box I). In addition, the two adjacent side arms indicated by Box II are still separated in Fig. 3b, while they have coalesced and entrapped a liquid droplet in Fig. 3c. The coalescence of adjacent secondary arms with entrapped liquid pockets can also be found in Fig. 3b (e.g. at the location indicated by an arrow). However, the entrapped liquid pockets maintain their irregular shapes rather than becoming nearly circular or elliptical as those in Fig. 3c.

**Figure 4:**
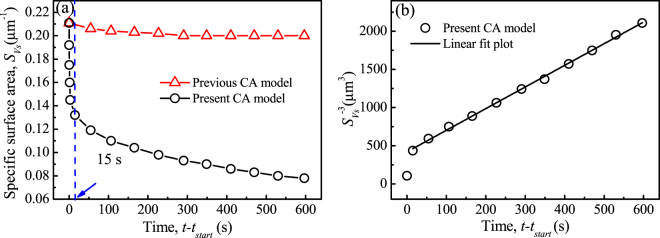
Evolution of specific surface area, *S*_*Vs*_, during isothermal holding in a mushy zone of 42.8 °C obtained from the previous and present CA models: (**a**) ${S}_{Vs}$ varying with time; (**b**) ${S}_{Vs}^{-3}$ varying with time.

As described in Section 1, the specific surface area is commonly employed to characterize dendrite coarsening. Figure 4a presents the profiles of the specific surface area, defined as the total S/L interfacial area per unit volume of solid, *S*_*Vs*_, varying with holding time, evaluated from the simulation results by the two CA models shown in Fig. 3b and c. It is noted that for the profile obtained from the previous CA model, *S*_*Vs*_ decreases slightly from the initial value of 0.211 μm^−1^ to about 0.2 μm^−1^ after holding for 600 s. For the profile obtained from the present CA model, however, *S*_*Vs*_ drops rapidly at the beginning from the initial *S*_*Vs*_ = 0.211 μm^−1^ to 0.132 μm^−1^ after holding for about 15 s. The initial fast drop in *S*_*Vs*_ is due to the fact that the dendritic microstructure prior to isothermal holding is simulated by the previous CA model. This model does not include the effect of melting/remelting and thus generates some very fine (highly curved) microstructural elements that disappear rapidly. Subsequently, *S*_*Vs*_ continuously decreases with a slower rate, but still faster than the curve from the previous CA model. After holding for 600 s, the value of *S*_*Vs*_ simulated by the present CA model decreases to 0.078 μm^−1^, about 2.6 times smaller than that simulated by the previous CA model. This shows clearly to what extent the remelting effect contributes to the coarsening process.

Numerous experimental studies showed that the specific surface area follows approximately a *t* ^−1/3^ power-law during dendrite coarsening^11–13,16,17^, even though the solid-liquid mixtures might not be evolving self-similarly^11,12^. Figure 4b shows the profile of ${S}_{Vs}^{-3}$ as a function of time, calculated from the present CA curve in Fig. 4a. The linear fitting plot, starting from *t* = *t*_*start*_ + 15 s, is also included. As shown, ${S}_{Vs}^{-3}$ varies approximately linearly with time.

**Figure 5:**
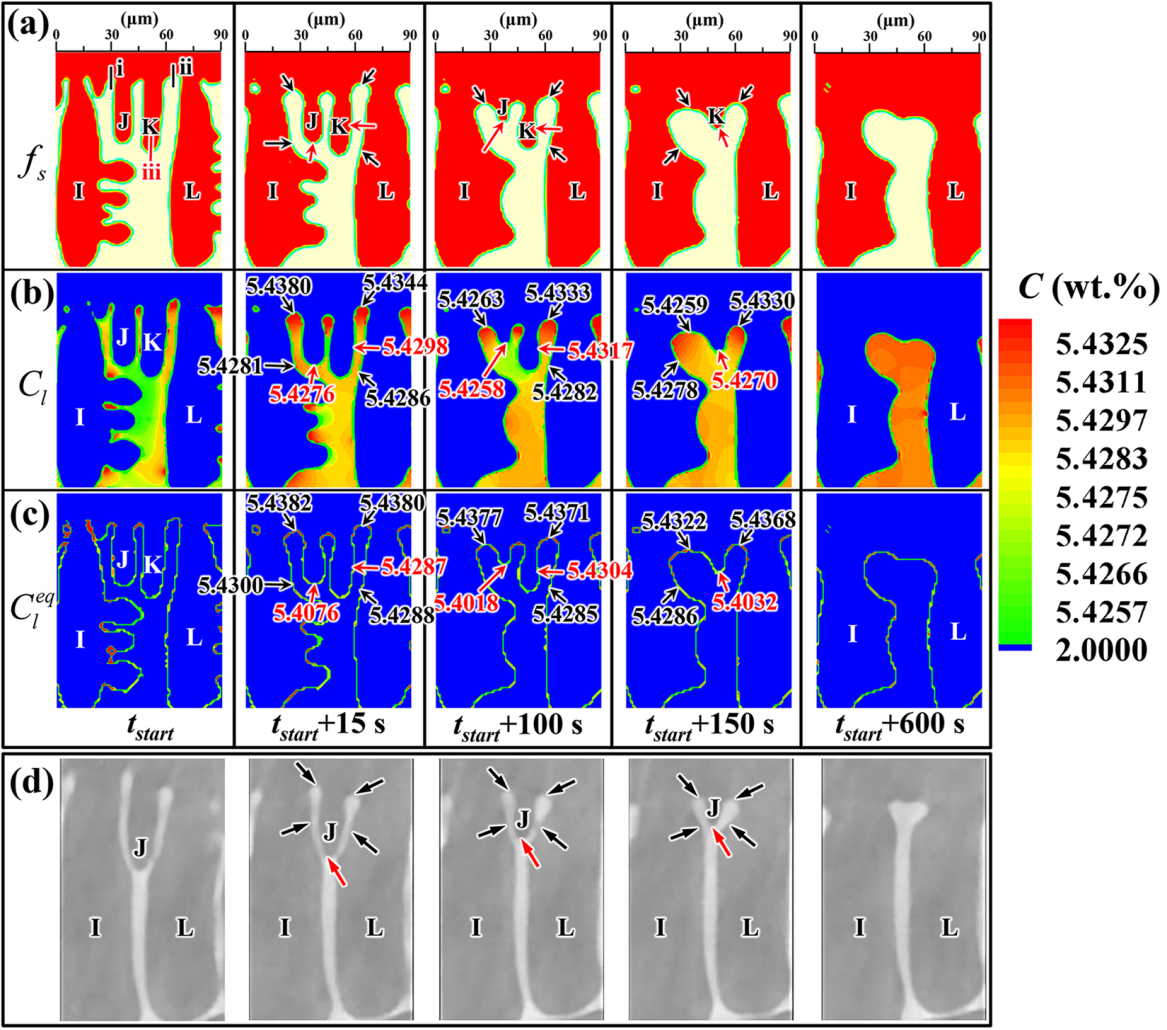
Evolution of dendrite coarsening simulated by the present CA model at the location of Box I in Fig. 3 during the holding time from *t*_*start*_ (Fig. 3a) to *t*_*start*_ + 600 s (Fig. 3c), displayed by the fields of (**a**) solid fraction; (**b**) actual composition; (**c**) equilibrium composition; (**d**) *in situ* experimental observations^9^. The numbers in the figures show (**b**) local actual liquid compositions, and (**c**) local equilibrium liquid compositions. The arrows and numbers in red color indicate melting locations, while the arrows and numbers in black color indicate solidification locations. The detailed evolution of the actual composition field shown in (**b**) can be seen in Supplementary video 2.

The typical features of dendrite coarsening observed in experiments^9^, such as small arm melting, interdendritic groove advancement, and arm coalescence, can be reproduced well by the present CA model, (see e.g. the regions of Boxes I and II in Fig. 3a and c). The microstructural evolution in the region of Box I during the holding time from *t*_*start*_ (Fig. 3a) to *t*_*start*_ + 600 s (Fig. 3c) is illustrated in Fig. 5 and analyzed in detail. Figure 5 presents the fields of (a) solid fraction, (b) actual composition, and (c) equilibrium composition. As shown, the main features include gradual dissolution of the small side arms ‘J’ and ‘K’, the bases of the interdendritic grooves gradually moving towards the arm tips, and the coalescence of fine tertiary arms by progressive filling of the interdendritic space, leading to a ‘swollen’ arm ‘I’.

Note that the local actual liquid compositions, *C*_*l*_, in the ‘valleys’ between side arms are higher than that at the tips of arms ‘J’ and ‘K’ as shown in Fig. 5b. If the curvature effect is not considered, the ‘valley’ regions should have higher tendency for melting than small arm tips. Yet, the variation of curvature leads to the variation of the equilibrium composition, ${C}_{l}^{eq}$. Apparently, the curvature is negative at the bases of the interdendritic grooves, while it is positive at arm tips. Calculated by Eq. (3), a lower curvature yields a higher ${C}_{l}^{eq}$. Accordingly, the local ${C}_{l}^{eq}$ in the interdendritic grooves is higher than that at the arm tips as shown in Fig. 5c. According to the equilibrium condition at the S/L interface (Eq. (6)), when *C*_*l*_ <${\,C}_{l}^{eq}$, solidification occurs (Δ*f*_*s*_ > 0), while the case of *C*_*l*_ >${\,C}_{l}^{eq}$ leads to melting (Δ*f*_*s*_ < 0). Comparing Fig. 5b and c, it is found that the conditions of *C*_*l*_ <${\,C}_{l}^{eq}$ and *C*_*l*_  > ${\,C}_{l}^{eq}$ are satisfied at the bases of the interdendritic grooves and the small arm tips, respectively. Therefore, it is understandable that the liquid grooves between side branches are gradually filled by local solidification, leading to the advancement of interdendritic grooves, while the small arms ‘J’ and ‘K’ are gradually shrinking and eventually disappear, being replaced by a liquid ‘trough’ between arms ‘I’ and ‘L’. Note that the condition of *C*_*l*_<$\,{C}_{l}^{eq}$ is also satisfied in the lateral side area of arms ‘I’ and ‘L’. Thus, thick arms ‘I’ and ‘L’ grow at the expense of the small arms ‘J’ and ‘K’.

It is known that solute atoms are rejected during solidification, but are absorbed during melting. Thus, a solute concentration gradient is established in the liquid phase, leading to solute transport from the solidifying to the remelting regions. As seen from Fig. 5b, the local compositions at the two grooves, where solidification occurs, slightly decrease during holding. Apparently, this is a result of solute diffusion from the interdendritic grooves to the vicinity of the small arms ‘J’ and ‘K’. On the one hand, the diffusion of solute atoms away from the S/L interfaces with relatively lower curvatures (e.g. interdendritic groove bases) promotes continuous solidification in those regions. On the other hand, the solute transport provides solute atoms to the vicinity of small side branches, facilitating the continuous remelting of those small arms. Consequently, Figure 5 clearly illustrates how remelting and solidification stimulate each other during the dendrite coarsening process.

In order to explore the influence of local remelting/solidification kinetics by the interfacial shapes in the vicinity, we have measured the net shrinkage length of the tip ‘K’ and the net growth lengths of the two grooves indicated by the red and black bars, respectively, in the first panel of Fig. 5a for different time intervals. As shown, during the time from *t*_*start*_ + 15 s to *t*_*start*_ + 100 s, arm ‘J’ is shrinking faster than arm ‘K’ that has a net shrinkage length of ∼3.5 μm in the position of Bar ‘iii’. When arm ‘J’ is almost dissolved, the shrinkage of arm ‘K’ is accelerated and yields a net shrinkage length of ∼9.5 μm during the time from *t*_*start*_ + 100 s to *t*_*start*_ + 150 s. Moreover, it is noted that the lateral sides of arms ‘K’ and ‘L’ are approximately straight, and thus the interfacial curvatures at those locations are both nearly zero. The right side of arm ‘K’, being close to the groove base, has a relatively higher *C*_*l*_, while at the left side of arm ‘L’, being close to the remelting tip of arm ‘K’, *C*_*l*_ is relatively lower. The conditions of *C*_*l*_ >${\,C}_{l}^{eq}$ and *C*_*l*_ <$\,{C}_{l}^{eq}$ are thus satisfied, leading to remelting and solidification, at the two side positions of arms ‘K’ and ‘L’, respectively. Therefore, in addition to the axial remelting of small arms, the approximately straight sides of arms also undergo the radial remelting/solidification depending on their environments. The radial remelting can thus not only occur due to the local curvature (which is finite in 3D, but approximately zero at a straight dendrite arm in 2D), but also due to the vicinity of solidifying microstructural features.

Then, let’s take a look at the two grooves indicated by Bars ‘i’ and ‘ii’ in the first panel of Fig. 5a. During the time from *t*_*start*_ + 15 s to *t*_*start*_ + 150 s, the net growth lengths of the two grooves are nearly identical around ∼11 μm. As shown in the fourth panels of Fig. 5, at the time of *t*_*start*_ + 150 s, arm ‘J’ has been completely dissolved, while the remaining arm ‘K’ is closer to the right groove. As a result, during the subsequent time from *t*_*start*_ + 150 s to *t*_*start*_ + 600 s, the net growth length of the right groove is around 9.5 μm that is about three times longer than that of the left groove. Accordingly, for studying the kinetics of dendrite coarsening, it is essential to analyze the complex interplay between the local interfacial shape and the morphologies of its neighboring arms, which considerably impacts the diffusion length and thereby the local actual liquid composition.

Figure 5d presents the progressive small arm remelting and interdendritic groove advancement observed by Terzi *et al*.^9^ using *in situ* X-ray tomography for an Al–10 wt.% Cu alloy held isothermally at 570 °C. Note that small arm ‘J’ is gradually melting until it is completely dissolved and replaced by a liquid ‘trough’ between arms ‘I’ and ‘L’. Because of local solidification, both arms ‘I’ and ‘L’ become thicker and the interdendritic groove base between the two arms gradually moves towards the tips.

**Figure 6:**
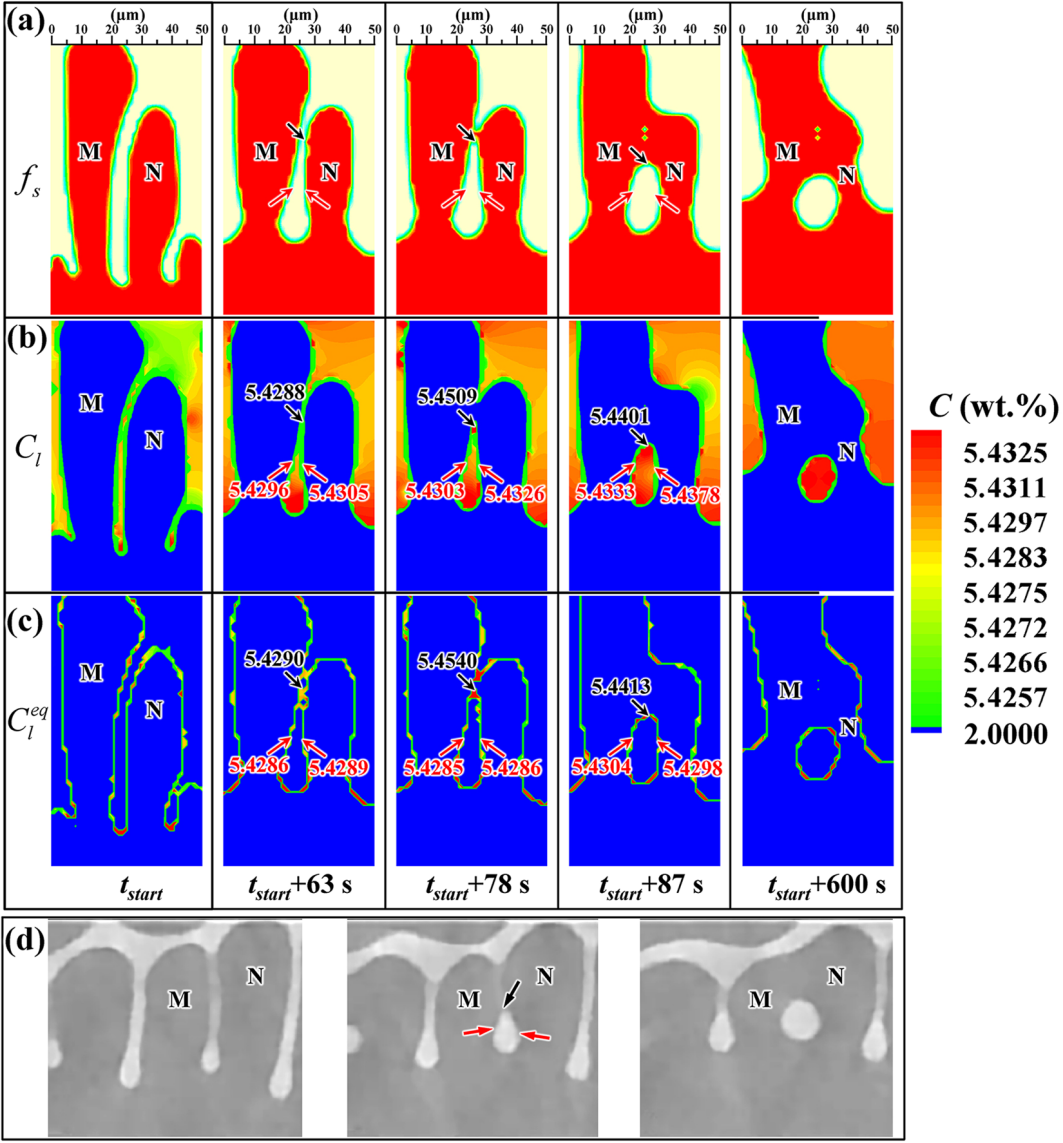
Evolution of dendrite coarsening simulated by the present CA model at the location of Box II in Fig. 3 during the holding time from *t*_*start*_ (Fig. 3a) to *t*_*start*_ + 600 s (Fig. 3c) displayed by the fields of (**a**) solid fraction; (**b**) actual composition; (**c**) equilibrium composition; (**d**) *in situ* experimental observations^9^. The numbers in the figures show (**b**) local actual liquid compositions, and (**c**) local equilibrium liquid compositions. The arrows and numbers in red color indicate melting locations, while the arrows and numbers in black color indicate solidification locations. The detailed evolution of the actual composition field shown in (**b**) can be seen in Supplementary video 3.

Figure 6 presents the microstructural evolution in the region of Box II in Fig. 3a and c. In addition to the interdendritic groove advancement, dendrite arm coalescence through joining arm tips can be observed. As shown in Fig. 6a, the side arms ‘M’ and ‘N’ gradually become coarser and then coalesce at the holding time of *t*_*start*_ + 78 s, leading to the entrapment of a liquid pocket with an irregular shape. In the following holding time, local solidification and remelting occur at the S/L interfaces of the top and two lateral sides of the entrapped liquid pocket, respectively. As a result, the entrapped liquid pocket is gradually transformed from an irregular shape to a nearly circular one. Figure 6b and c present the local actual liquid compositions, *C*_*l*_, and equilibrium liquid compositions, ${C}_{l}^{eq}$, at different locations. As shown, the conditions of *C*_*l*_ <$\,{C}_{l}^{eq}$ and *C*_*l*_ >${\,C}_{l}^{eq}$ are fulfilled at the top and two lateral sides of the liquid droplet, respectively, providing a straightforward explanation of the local remelting and solidification phenomena.

Figure 6d presents the progressive joining of the tips of the dendrite arms, leading to the formation of an entrapped liquid droplet in the solid, as experimentally observed by Terzi *et al*.^9^ under a same condition as those in Fig. 5d. As shown in Figs 5 and 6, the coarsening features of dendritic microstructures during isothermal holding in a mushy zone simulated by the present CA model (Figs 5a–c and 6a–c) compare well with the *in situ* experimental observations (Figs 5d and 6d).

**Figure 7:**
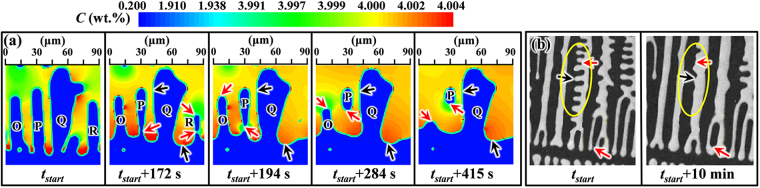
Evolution of dendrite arm fragmentation: (**a**) simulated by the present CA model and shown by the field of local actual composition; (**b**) *in situ* experimental observations^15^. The arrows in red and black colors indicate melting and solidification locations, respectively. The detailed evolution of dendrite arm fragmentation shown in (**a**) can be seen in Supplementary video 4.

As summarized by Flemings *et al*.^10^, dendrite arm fragmentation is one of the important dendrite coarsening mechanisms. A neck may form at the root of a small side arm surrounded by larger arms. By further remelting at the neck of the arm and solidification at the neighboring sites that have lower curvatures, the small side arm is detached from the main dendrite stem^10^. This phenomenon has also been observed in the simulations of isothermal dendrite coarsening using the present CA model. Figure 7a presents the simulated evolution of dendrite arm fragmentation for a SCN-2.0wt.%ACE alloy during isothermal holding at 46.9 °C, which is a small region taken from the calculation domain of a 140 × 600 mesh with Δ*x* = 1.2 μm. Other simulation conditions are identical with those of Fig. 3. The arrows in black and red colors indicate the solidification and melting locations, respectively. As seen in Fig. 7a that displays the local actual composition field, the large secondary arm ‘Q’ is initially surrounded by four small arms. After isothermal holding for 172 s, the small side branch between arms ‘Q’ and ‘R’ disappears, while arm ‘Q’ becomes thicker and the interdendritic groove base moves towards the arm tips. These features are identical with the coarsening phenomena shown in Fig. 5. At the time of *t*_*start*_ + 172 s, the small arm ‘R’ has detached from the primary dendrite stem and a neck forms at the root of arm ‘P’. Note that at *t*_*start*_ + 172 s the local liquid actual composition around the root of arm ‘P’ is apparently higher than that in the liquid near the arm tips. This is because the freezing at the interdendritic groove base and at the large arm ‘Q’ rejects solute atoms, leading to solute enrichment in the vicinity of arm roots. At *t*_*start*_ + 194 s, the detachment of arm ‘P’ occurs and arm ‘R’ has completely disappeared. In the period from *t*_*start*_ + 194 s to *t*_*start*_ + 284 s, the remelting of the small arm ‘O’ occurs, consuming solute atoms in the liquid near arm ‘P’, which reduces the remelting rate of arm ‘P’. After arm ‘O’ is nearly dissolved, the remelting of arm ‘P’ is found to speed up and it gradually spheroidizes. Figure 7b presents the evolution of dendrite arm fragmentation obtained by Jackson *et al*.^15^ using the *in situ* observation experiments with a transparent cyclohexanol-fluorescein alloy. As shown, in addition to the remelting of the small tertiary arms to the benefit of the large secondary arms, the side arm detachment at the root can be clearly observed.

It can be seen from the first panels of Figs 5b and 6b, and 7a that there is a certain level of microsegregation in the microstructures at time *t*_*start*_, which is inherited from the pre-coarsening cooling stage. There will certainly be an influence on the subsequent dendrite coarsening process. It is well known that alloy composition and solidification rate will significantly impact both dendrite morphology and microsegregation^17,45^. In the present work, the alloy composition and solidification cooling conditions are identical for all simulation results of Figs 3–7. The influence of the initial dendrite morphology and microsegregation prior to the isothermal holding is not discussed in detail.

### Analysis of solidification and remelting in 2-D versus 3-D

The analysis of dendrite coarsening in Figs 5 and 6 uses comparisons of the local actual composition determined by mass transfer and the local equilibrium composition associated with the local curvature. Thus, the isothermal coarsening process is controlled by solute diffusion and the variation of interfacial curvature. In this section, we analyze the kinetics of solidification/remelting in 2-D versus 3-D through the discussion regarding the similarity and difference between 2-D and 3-D for the interfacial curvature distribution and solute diffusion during the isothermal dendrite coarsening process.

**Figure 8:**
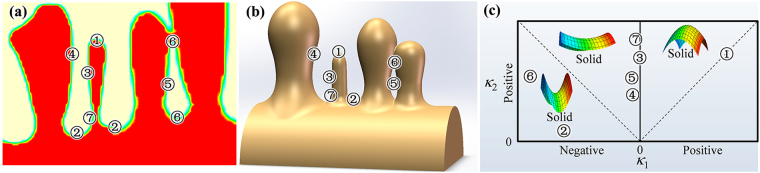
Map of local principle curvatures at different locations of a dendrite in 2-D and 3-D: schematic drawing of a section of a primary dendrite stem with four arms in (**a**) 2-D; (**b**) 3-D; (**c**) map of local interfacial shapes with different principle curvatures for the locations indicated by the numbers in (**b**).

The local interfacial curvature in 3-D is given by *K*_3*D*_ = *κ*_1_ + *κ*_2_, where *κ*_1_ and *κ*_2_ are the two local principal curvatures^46^. In Fig. 8a and b the interfacial locations tagged with different numbers correspond to different locations in Figs 5a–c and 6a–c and 7a. Apparently, the curvatures calculated by Eq. (4) in 2-D can be taken as the local first principal curvatures, *κ*_1_. However, the second principal curvatures, *κ*_2_, that are in the sections perpendicular to the page plane need to be qualitatively estimated. Figure 8c presents a map of different *κ*_1_ and *κ*_2_ for the different locations indicated in Figs 5a–c and 6a–c and 7a. In the following we analyze the local curvatures in 3-D for those locations.

(1) The location tagged with ‘1’ corresponds to the small arm tips in Figs 5a–c and 7a. For symmetrical arm tips, the two principal curvatures are positive and identical, i.e., *κ*_2_(1) = *κ*_1_(1). The local curvature in 3-D is *K*_3*D*_(1) = 2*κ*_1_(1). As stated above, the 2-D local curvature calculated by Eq. (4) is taken as *κ*_1_. Thus, the local curvature in 3-D is larger than that in 2-D for small arm tips.

(2) The location tagged with ‘2’ corresponds to the liquid groove bases between the secondary arms in Figs 5a–c and 7a, which are saddle-shaped patches in 3-D. The first principal curvature, *κ*_1_(2), is highly negative, while the second principal curvature is positive *κ*_2_(2) = 1/*R*_*p*_, where *R*_*p*_ is the radius of the primary dendrite stem. Considering that *R*_*p*_ is much larger than the groove radius, it is justified to neglect *κ*_2_(2). Thus, the 3-D curvature at the location ‘2’ would still be negative, but it is slightly larger than that in 2-D.

(3) The locations tagged with ‘3’ and ‘4’ correspond to the lateral sides of small and large arms in Figs 5a–c and 7, respectively. The first principal curvatures, *κ*_1_(3) and *κ*_1_(4), are approximately zero, while the second principal curvatures, being the inverse side arm radius, are positive with *κ*_2_(3) > *κ*_2_(4) > 0. Thus, the 3-D curvatures, *K*_3*D*_(3) ≈ *κ*_2_(3) > *K*_3*D*_(4) ≈ *κ*_2_(4) > 0, are larger than those in 2-D. Note that when only considering the curvature effect, the driving force for radial remelting/solidification of small and large side arms should be nearly identical in 2-D, while it is different in 3-D, due to the different 3-D curvatures caused by the different arm radii. Nevertheless, as discussed above, the kinetics of remelting/solidification is not only related to the local curvature, but also influenced significantly by the environment. The radial remelting of small arms and the growth of thick arms have also been observed in the 2-D simulation, depending on their neighboring shapes as shown in Figs 5 and 7.

(4) The locations tagged with ‘5’ and ‘6’ correspond to the lateral sides and the top (bottom) of the entrapped liquid droplet in Fig. 6a–c, respectively; both are saddle-shaped in 3-D. The first principal curvature at location ‘5’, *κ*_1_(5), is slightly negative, while *κ*_1_(6) is highly negative. The second principal curvatures of the two locations *κ*_2_(5) and *κ*_2_(6) are both positive. The relation is |*κ*_1_(5)| < *κ*_2_(5)≈*κ*_2_(6) < |*κ*_1_(6)|. Thus, the 3-D curvature at location ‘5’ is overall positive (*K*_3*D*_(5) > 0), while it is overall negative at location ‘6’ (*K*_3*D*_(6) < 0).

(5) The location tagged with ‘7’ corresponds to the lateral sides at the root of small arms in Fig. 7a. The first principal curvature, *κ*_1_(7), is approximately zero, while the second principal curvature is highly positive, due to the small side arm radius at the necked root. Thus, the 3-D curvature, *K*_3*D*_(7) ≈ *κ*_2_(7) > 0, is larger than that in 2-D.

Therefore, the 3-D local curvatures of different locations shown in Fig. 8b follow the relation *K*_3*D*_(2) < *K*_3*D*_(4) < *K*_3*D*_(3) < *K*_3*D*_(7) < *K*_3*D*_(1) and *K*_3*D*_(6) < *K*_3*D*_(5), which is nearly the same as in 2-D. However, the curvatures 2-D are generally somewhat smaller than that in 3-D.

It is well known that solute atoms diffuse from the locations with lower curvatures to those with higher curvatures. As analyzed above, the interfacial curvature distributions in 2-D are similar to those in 3-D. Thus, the solute diffusion directions in 2-D and 3-D are essentially the same, e.g. from the interdendritic groove bases and lateral sides of large arms (‘2’ and ‘4’) to the tips, lateral sides and roots of small side arms (‘1’, ‘3’ and ‘7’), and from the top and bottom locations (‘6’) to the lateral sides (‘5’) of the entrapped liquid droplet. It is noted, however, that the solute diffusion in 3-D can be more effective than that in 2-D even under the condition of identical solute concentration gradients, due to the different diffusion spaces in 2-D and 3-D^35,47^.

Accordingly, it is expected that the 2-D model can yield the same trend of local solidification/remelting as in 3-D, due to the similarities of interfacial curvature distributions and solute diffusion directions in 2-D and 3-D. As presented in Figs 5, 6, and 7, the present 2-D CA simulations can qualitatively reproduce most dendrite coarsening phenomena observed in 3-D experiments. Because of differences in the quantity of local interfacial curvatures and diffusion spaces in 2-D and 3-D, the kinetics of local solidification/remelting may slightly deviate in 2-D and 3-D.

## Conclusions

A 2-D CA model is proposed for the simulation of microstructural evolution involving both solidification and melting in mushy zones of alloys. The present model is an extension of a previous CA model by incorporating local melting. The kinetics of the S/L interface migration is calculated according to the difference between the local equilibrium and actual liquid compositions. The local equilibrium liquid composition is associated with the local temperature and weighted curvature incorporated with the anisotropy of surface energy, while the actual liquid composition is determined by solute diffusion.

The model validation is performed by a comparison of the CA simulation with the analytical solution for the migration of a liquid pool in the mushy zone of a SCN–0.3 wt.% ACE alloy due to TGZM. In a static temperature gradient, the liquid pool migrates towards the liquidus and its migration velocity gradually increases with time. The simulated pool position and migration velocity varying with time are in good agreement with the analytical predictions.

Dendrite coarsening of a SCN–2.0 wt.% ACE alloy during isothermal holding in a mushy zone is simulated using the previous and present CA models. After holding for 600 s, the microstructures simulated by the two models are considerably different. The specific surface area, *S*_*Vs*_, obtained from the present CA model is about 2.6 times smaller than that from the previous CA model, showing how remelting impacts the coarsening process. Typical coarsening features, such as the dissolution of small side arms to the benefit of their neighbours, advancement of interdendritic groove bases, coalescence of adjacent arms, and dendrite arm fragmentation are observed in the microstructures simulated by the present model, which compares fairly well with the *in situ* experimental observations reported in the literature. Solidification occurs at the locations with a higher equilibrium composition that results from the lower weighted curvature of the concave interface (e.g. the bases of interdendritic grooves). In contrast, the large weighted curvature of the convex interface (e.g. the tips of small arms) produces a relatively lower equilibrium composition, leading to local remelting. The approximately straight sides of the secondary arms might also undergo the radial remelting/solidification depending on their environments. In addition, owing to the local solute enrichment, the root of a small side arm surrounded by larger arms may become necked and then detach from the main stem. It is quantified how the kinetics of local solidification/remelting is not only associated with the local curvature, but also significantly influenced by the shapes of its neighboring interfaces, which remarkably impacts the diffusion length and thereby the local actual liquid composition. The analysis of interfacial curvature distribution and solute diffusion in 3-D yields the same trend of local solidification/remelting as those analyzed using the results by the 2-D CA simulations. The simulation results by the present CA model provide insight into the complicated interactions among the kinetics of local solidification/remelting, interfacial shape variation, and solute diffusion during dendrite coarsening process.

### Data availability

All data generated or analysed during this study are included in this published article (and its Supplementary Information files).

## Electronic supplementary material

supplementary video 1

supplementary video 2

supplementary video 3

supplementary video 4
